# Voice disorder is recognized as an occupational disease

**DOI:** 10.1590/2317-1782/e20250357en

**Published:** 2026-06-19

**Authors:** Maria Lúcia Vaz Masson, Léslie Piccolotto Ferreira, Claúdia Giglio de Oliveira Gonçalves

**Affiliations:** 1 Programa de Pós-graduação em Ciências da Reabilitação – PPG-REAB, Departamento de Fonoaudiologia, Instituto Multidisciplinar de Reabilitação e Saúde – IMRS, Universidade Federal da Bahia – UFBA - Salvador (BA), Brasil.; 2 Programa de Pós-graduação em Saúde, Ambiente e Trabalho – PPGSAT, Faculdade de Medicina da Bahia – FAMEB, Universidade Federal da Bahia – UFBA - Salvador (BA), Brasil.; 3 Grupo de Trabalho Agravos da Comunicação Relacionados ao Trabalho – GT-DVRT/PAIR, Sociedade Brasileira de Fonoaudiologia – SBFa - São Paulo (SP), Brasil.; 4 Programa de Pós-graduação em Comunicação Humana e Saúde, Departamento de Teorias e Métodos da Fonoaudiologia e da Fisioterapia, Pontifícia Universidade Católica de São Paulo – PUC-SP - São Paulo (SP), Brasil.; 5 Fórum das Entidades Nacionais dos Trabalhadores da Área de Saúde – FENTAS, Conselho Nacional de Saúde – CNS - Brasília (DF), Brasil.

On the agenda and in the name of the ***Work-Related Voice Disorders Working Group (GT-DVRT/PAIR)*** of the Brazilian Society of Speech-Language Pathology and Audiology (SBFa), we would like to announce the official recognition of voice disorder (VD) as a work-related condition by the Ministry of Health (MoH), in a historic redress that recovers dimensions that had been neglected until then in the field of Voice.

This achievement means an equitable response, with epidemiological guidance, for vulnerable groups that most need care for the voice (teachers, teleoperators, among others). Since the first concerns regarding a high demand from teachers at a public hospital in Recife (PE), academic seminars were organized, debates with unions, professional bodies, and social actors^(1)^, in parallel with research^(2,3)^ for the production of evidence on Work-Related Voice Disorder (WRVD), which culminated in the publication of the WRVD Protocol^(4)^.

The main question that arises is the absence of information about the environment and work organization in studies that address vocal health, especially among teachers, which makes it difficult to establish the causal link^(5)^, an essential aspect in defining a work-related condition. Most of the time, the worker is blamed for their illness when, in reality, it is an imposition of work, such as the intense voice use by teachers^(6)^. It is no coincidence that, despite numerous studies, teachers continue to fall ill.

In this letter, we highlight four fundamental points: 1) the publication of the Differentiated Complexity Protocol – WRVD^(4)^; 2) the inclusion of voice disorder in the List of Work-Related Diseases (LWRD)^(7)^; 3) the inclusion of WRVD in the National List of Notifiable Conditions (NLNC)^(8)^; and 4) the publication of the technical norm with guidelines for WRVD Surveillance^(9)^.

## DIFFERENTIATED COMPLEXITY PROTOCOL – WRVD

The WRVD Protocol^(4)^ was the first official document, published by the MoH, and it brings together information on epidemiology, prevention, risk factors, assessment, diagnosis, and treatment, in an integrated care network, with promotion, protection, and rehabilitation actions, with a focus on Workers' Health Surveillance (WHS). The importance of WHS is established in its capacity to intervene in environments and work processes, in order to promote healthy environments and prevent diseases and health conditions^(10)^.

It should be highlighted that, in addition to the well-known voice professionals (among them teachers) exposed to vocal overload, the Protocol included other occupations, understanding that exposure to risk factors arising from the environment and work organization processes can compromise people's voice and communication.

## LIST OF WORK-RELATED DISEASES (LWRD)

The LWRD^(9)^ has a broad perspective, whose objective is to guide the clinical-epidemiological use of diseases and conditions, aiming to offer comprehensive actions to Workers' Health, facilitate the study of the relationship between illness and work, adopt diagnostic procedures, develop therapeutic projects, and guide surveillance and health promotion actions at individual and collective levels.

The first LWRD^(11)^, systematized in the Manual of Work-Related Diseases^(12)^, did not include VD. The revision of the LWRD took place in the midst of the COVID-19 pandemic scenario, when a technical committee organized the process, including a national public consultation. In an arbitrary act, the ordinance was revoked, leaving a normative gap, without legislation to support public policies aimed at Workers' Health^(13)^. After negative reactions from society, the Public Labor Prosecutor's Office issued a note stating that the revocation was an “undeniable political and legal mistake”, a measure against the health and life of working people^(14)^. The insecurity arising from the revocation evidenced a “political imbroglio”^(15)^, characterized by instability in ministerial leadership, with the old list being reinstated, that is, returning to force without the inclusion of new diseases^(16)^.

Only in the current government was WRVD included in the LWRD with the main code R49 – Voice Disorders, according to the International Classification of Diseases (ICD-10), expressed in Chapter X – Diseases of the Respiratory System, characterized by the codes J37.1 - Chronic laryngotracheitis, J38 - Diseases of the vocal cords and larynx not classified elsewhere, J38.2 - Vocal cord nodules; and R49 itself, present in the Chapter XVIII - Symptoms and Signals ^(7)^.

An updated version was published in 2024^(17)^, including chemical risk factors (bromine, iodine, caustic and toxic agents) and inserting the code J04.2 - Acute laryngotracheitis, in order to map cases at the beginning of exposure.

## NATIONAL LIST OF NOTIFIABLE CONDITIONs (NLNC)

While the LWRD^(7,11,17)^ in its different versions is larger and directed toward public health policies, the NLNC^(8)^ is reduced and aims to establish a model of universal surveillance in Workers' Health.

The inclusion of WRVD in the NLNC allows the identification and intervention in environments and work processes, contributing directly to the improvement of quality of life, in addition to reducing social and economic costs associated with occupational conditions. Notification must be carried out upon suspicion of the condition, in a compulsory (mandatory) manner, on the single registration platform e-SUS Sinan^(9)^ (Brazilian Case Registry Database), with the speech-language pathologist being qualified to notify speech-language disorders.

## TECHNICAL NOTE - WRVD SURVEILLANCE

In Technical Note No. 21/2025^(9)^, the MoH organized, in a single document, guidelines for the National Network of Comprehensive Care for Workers' Health (Renast) on how to handle WRVD. The WHS process is presented in five stages in this Note: 1) case identification; 2) compulsory notification in e-SUS Sinan; 3) epidemiological investigation for analysis of the relationship with work and collective risk situations; 4) monitoring and continuous analysis of data and risk situations; and 5) intervention on exposure factors, conditioning and environmental, organizational, and psychosocial determinants of the condition.

With the data fed into a single platform (e-SUS Sinan), monitored and analyzed continuously, there will be greater clarity on the situations and places where the risk presents itself, with interventions proposed to promote vocal health and prevent dysphonia among workers.

It is worth highlighting that any health professional, such as the speech-language pathologist, must notify WRVD, including private services. If the clinic does not have registration in the CNES (National Registry of Health Facilities), it may associate, upon authorization, with a registered service^(18)^. To do so, it is necessary to access e-SUS Sinan^(19)^ and request registration, identifying the appropriate profile and including a justification^(20)^. In the recent Webinar^(18)^, the MoH guided step by step how to perform registration and WRVD notification on the platform^(18)^.

In addition to the historic record on the recognition of WRVD, it is important that the reader, especially the speech-language pathologist, understand how these advances can change their practice, whether regarding assessment procedures for a worker with vocal complaints, the distinction between a common VD and a WRVD, and the importance of notification which, if not carried out, will cause a scarcity of reliable data to support public policies. To support this knowledge, we suggest reading references that can detail issues concerning the recognition of WRVD^(6,21,22)^, its understanding in the field of Workers' Health^(4,23)^, and its comprehension according to the International Classification of Functioning (ICF)^(24)^.

Finally, although happy with the achievements, we have key challenges ahead, such as the implementation of the WRVD care network, for which we need the participation and engagement of speech-language pathologists, whether from the public network or private services. We count on you in this endeavor!

## References

[1] Ferreira LP, Bernardi APA (2011). Distúrbio de voz relacionado ao trabalho: resgate histórico. Distúrb Comum.

[2] Ferreira LP, Oliveira SMRP (2004). Voz profissional: produção científica da fonoaudiologia brasileira..

[3] Ferreira LP, Giannini SPP, Figueira S, Silva EE, Karmann DF, Souza TMT (2003). Condições de produção vocal de professores da Prefeitura do Município de São Paulo. Distúrb Comum.

[4] Brasil (2018). Distúrbio de voz relacionado ao trabalho - DVRT.

[5] Ribeiro BC (2024). Nexo causal entre trabalho e saúde/doença e o problema das perícias. Rev Bras Saude Ocup.

[6] Masson MLV, Ferrite S, Pereira LMA, Ferreira LP, Araújo TM (2019). Em busca do reconhecimento do distúrbio de voz como doença relacionada ao trabalho: movimento histórico-político. Cien Saude Colet.

[7] Brasil (2023). Portaria GM/MS nº 1.999, de 27 de novembro de 2023. Altera a Portaria de Consolidação GM/MS nº 5, de 28 de setembro de 2017, para atualizar a Lista de Doenças Relacionadas ao Trabalho (LDRT).

[8] Brasil (2024). Portaria GM/MS nº 5.201, de 15 de agosto de 2024. Altera o Anexo 1 do Anexo V à Portaria de Consolidação MS nº 4, de 28 de setembro de 2017, para incluir novas doenças na Lista Nacional de Notificação Compulsória.

[9] Brasil (2025). Nota Técnica nº 21/2025 – CGSAT/DVSAT/ SVSA/MS. Orientações para a vigilância do Distúrbio de Voz Relacionado ao Trabalho (DVRT).

[10] Hurtado SLB, Silva-Macaia AA, Vilela RAG, Querol MAP, Lopes MGR, Bezerra JLC (2022). Intervenções em saúde do trabalhador - contexto, desafios e possibilidades de desenvolvimento: uma revisão de escopo. Rev Bras Saude Ocup.

[11] Brasil (1999). Portaria GM/MS nº 1.339, de 18 de novembro de 1999. Institui a Lista de Doenças Relacionadas ao Trabalho, a ser adotada como referência dos agravos originados no processo de trabalho no Sistema Único de Saúde.

[12] Brasil (2001). Doenças relacionadas ao trabalho: manual de procedimentos para os serviços de saúde.

[13] Brasil (2020). Portaria GM/MS nº 2.345, de 2 de setembro de 2020. Torna sem efeito a Portaria nº 2.309/GM/MS, de 28 de agosto de 2020.

[14] Brasil (2020). Nota pública sobre a revogação da Lista de Doenças Relacionadas ao Trabalho (LDRT).

[15] Dias EC, Silva-Junior JS, Baeta KF, Bandini M (2021). Lista de doenças relacionadas ao trabalho: obrigação legal de base técnica se transforma em imbróglio político-social: reflexões sobre possíveis saídas. Saude Debate.

[16] Brasil (2020). Portaria GM/MS nº 2.384, de 9 de setembro de 2020. Repristina os arts. 423 e 424 da Seção IV do Capítulo III do Título III e o Anexo LXXX da Portaria de Consolidação nº 5/GM/MS, de 28 de setembro de 2017.

[17] Brasil (2024). Portaria GM/MS nº 5.674, de 1º de novembro de 2024. Atualiza a Lista de Doenças Relacionadas ao Trabalho (LDRT), constante da Portaria de Consolidação GM/MS nº 5, de 28 de setembro de 2017.

[18] Brasil (2025). Avanços, perspectivas e desafios: a vigilância em saúde do trabalhador e da trabalhadora.

[19] Brasil (2025). e-SUS Sinan.

[20] Brasil (2025). Procedimento Operacional Padrão: manuseio e registro de doenças de notificação/conclusão no e-SUS Sinan.

[21] Masson MLV, Ferreira LP, Maeno M (2024). Distúrbio de voz relacionado ao trabalho: um olhar sobre o passado, o presente e o futuro. Rev Bras Saude Ocup.

[22] Masson MLV, Ferreira LP, Giannini SPP, Souza MT, Maeno M, Gândara MER (2020). Distúrbio de voz: reconhecimento revogado junto com a nova lista de doenças relacionadas ao trabalho. Rev Bras Saude Ocup.

[23] Ferreira LP, Andrada e Silva MA (2022). Distúrbio de voz relacionado ao trabalho: conquistas e desafios na América Latina.

[24] Biz MC, Masson ML, Souza MT, Ferreira LP, Paiva SF, Pinto FCA (2023). Classificação Internacional de Funcionalidade: da teoria à prática em Fonoaudiologia..

