# Supplementary material for: Voice disorder is recognized as an occupational disease
Source: Codas. 2026 Jun 19;38(4):e20250357. doi: 10.1590/2317-1782/e20250357en (PMC13322484; doi:10.1590/2317-1782/e20250357en)
Supplement: Translation

# Translation: O distúrbio de voz é reconhecido como doença relacionada ao trabalho

**DOI:** 10.1590/2317-1782/e20250357pt

**Authors:** Maria Lúcia Vaz Masson, Léslie Piccolotto Ferreira, Claúdia Giglio de Oliveira Gonçalves

**Affiliations:** 1 Programa de Pós-graduação em Ciências da Reabilitação – PPG-REAB, Departamento de Fonoaudiologia, Instituto Multidisciplinar de Reabilitação e Saúde – IMRS, Universidade Federal da Bahia – UFBA - Salvador (BA), Brasil.; 2 Programa de Pós-graduação em Saúde, Ambiente e Trabalho – PPGSAT, Faculdade de Medicina da Bahia – FAMEB, Universidade Federal da Bahia – UFBA - Salvador (BA), Brasil.; 3 Grupo de Trabalho Agravos da Comunicação Relacionados ao Trabalho – GT-DVRT/PAIR, Sociedade Brasileira de Fonoaudiologia – SBFa - São Paulo (SP), Brasil.; 4 Programa de Pós-graduação em Comunicação Humana e Saúde, Departamento de Teorias e Métodos da Fonoaudiologia e da Fisioterapia, Pontifícia Universidade Católica de São Paulo – PUC-SP - São Paulo (SP), Brasil.; 5 Fórum das Entidades Nacionais dos Trabalhadores da Área de Saúde – FENTAS, Conselho Nacional de Saúde – CNS - Brasília (DF), Brasil.

Na ordem do dia e em nome do ***Grupo de Trabalho Agravos da Comunicação Relacionados ao Trabalho (GT-DVRT/PAIR)*** da Sociedade Brasileira de Fonoaudiologia (SBFa), gostaríamos de anunciar o reconhecimento oficial do distúrbio de voz (DV) como agravo relacionado ao trabalho pelo Ministério da Saúde (MS), num reparo histórico que resgata dimensões até então negligenciadas na área de Voz.

A importância dessa vitória é oferecer uma resposta equitativa, com orientação epidemiológica, a grupos vulnerabilizados que mais necessitam de cuidado com a voz (professores, teleoperadores, entre outros). Desde as primeiras inquietações a respeito de uma elevada demanda de professores num hospital público de Recife (PE), organizaram-se seminários acadêmicos, debates com sindicatos, órgãos de classe e atores sociais^(1),^ em paralelo com pesquisas^(2,3)^ para produção de evidências sobre o Distúrbio de Voz Relacionada ao Trabalho (DVRT), que culminaram com a publicação do Protocolo DVRT^(4)^

A questão principal que se coloca é a ausência de informações sobre o ambiente e a organização do trabalho nos estudos que abordam a saúde vocal, especialmente de docentes, o que dificulta o estabelecimento do nexo causal^(5),^ aspecto essencial para a definição de um agravo relacionado ao trabalho. Na maioria das vezes, o/a trabalhador/a é culpabilizado pelo seu adoecimento quando, na verdade, é uma imposição do trabalho, como o uso intenso da voz por professores/as^(6)^. Não é à toa que, a despeito de inúmeras pesquisas, os/as professores/as continuam adoecendo.

Nesta carta, destacamos quatro pontos fundamentais: 1) a publicação do Protocolo de Complexidade Diferenciada – DVRT^(4)^; 2) a inclusão do distúrbio da voz na Lista de Doenças Relacionadas ao Trabalho (LDRT)^(7)^; 3) a inclusão do DVRT na Lista Nacional de Notificação Compulsória (LNNC)^(8)^; e 4) a publicação da norma técnica com orientações para a Vigilância do DVRT^(9)^.

## PROTOCOLO DE COMPLEXIDADE DIFERENCIADA – DVRT

O Protocolo DVRT^(4)^ foi o primeiro documento oficial, publicado pelo MS e agrega informações sobre epidemiologia, prevenção, fatores de risco, avaliação, diagnóstico e tratamento, numa rede de cuidado integral, com ações de promoção, proteção e reabilitação, com foco na Vigilância à Saúde do Trabalhador (Visat). A importância da Visat se estabelece na capacidade de intervir em ambientes e processos de trabalho, de modo a promover condições saudáveis e prevenir doenças e agravos à saúde^(10)^.

Deve-se destacar que, além dos/as conhecidos/as profissionais da voz (dentre eles/elas os/as professores/as) expostos/as à sobrecarga vocal, o Protocolo incluiu outras ocupações, entendendo que a exposição a fatores de risco advindos do ambiente e dos processos de organização do trabalho pode comprometer a voz e a comunicação das pessoas.

## LISTA DE DOENÇAS RELACIONADAS AO TRABALHO (LDRT)

A LDRT^(9)^ tem uma perspectiva ampla, cujo objetivo é orientar o uso clínico-epidemiológico de doenças e agravos, visando ofertar ações integrais à saúde do/a trabalhador/a, facilitar o estudo da relação entre adoecimento e trabalho, adotar procedimentos diagnósticos, elaborar projetos terapêuticos e orientar as ações de vigilância e promoção da saúde em níveis individual e coletivo.

A primeira LDRT^(11)^, sistematizada no Manual de Doenças Relacionadas ao Trabalho^(12)^, não incluía o DV. A revisão da LDRT ocorreu em pleno cenário pandêmico da COVID-19, quando uma comissão técnica organizou o processo, incluindo uma consulta pública nacional. Num ato arbitrário, a portaria foi revogada, criando um vácuo normativo, sem legislação que sustentasse as políticas públicas voltadas à Saúde do/a Trabalhador/a^(13)^. Após reações negativas da sociedade, o Ministério Público do Trabalho lançou uma nota afirmando que a revogação foi um “inegável equívoco político e jurídico”, numa medida contrária à saúde e à vida das pessoas que trabalham^(14)^. A insegurança decorrente da revogação evidenciou um “imbróglio político”^(15)^, caracterizado pela instabilidade da condução ministerial, sendo a antiga lista repristinada, ou seja, voltando a vigorar sem a inclusão das novas doenças^(16)^.

Somente no governo atual o DVRT foi incluído na LDRT com o código principal R49 – Distúrbios da Voz, conforme a Classificação Internacional das Doenças (CID-10), expresso no Capítulo X – Doenças do Aparelho Respiratório, caracterizado pelos CIDs J37.1 - Laringotraqueíte crônica, J38 - Doenças das cordas vocais e da laringe não classificadas em outra parte, J38.2 - Nódulos das cordas vocais; e o próprio R49, presente Capítulo XVIII - Sintomas, Sinais e achados anormais^(7)^.

Em 2024, foi publicada uma versão atualizada^(17)^, incluindo fatores de risco químicos (bromo, iodo, agentes cáusticos e tóxicos) e foi inserido o código J04.2 - Laringotraqueíte aguda, de modo a mapear casos no início da exposição.

## LISTA NACIONAL DE NOTIFICAÇÃO COMPULSÓRIA (LNNC)

Enquanto a LDRT^(7,11,17)^, nas suas diferentes versões, é maior e direcionada a políticas públicas de saúde, a LNNC^(8)^ é reduzida e tem por finalidade instituir um modelo de vigilância universal em Saúde do/a Trabalhador/a.

A inclusão do DVRT na LNNC permite identificar e intervir em ambientes e processos de trabalho, contribuindo diretamente para a melhoria da qualidade de vida, além de reduzir custos sociais e econômicos associados aos agravos ocupacionais. A notificação deve ser realizada na suspeita do agravo, de maneira compulsória (obrigatória), na plataforma de registro único e-SUS Sinan^(9)^ (Sistema Nacional de Notificação de Doenças), sendo o/a fonoaudiólogo/a apto/a para notificá-lo.

## NOTA TÉCNICA - VIGILÂNCIA DO DVRT

Na Nota Técnica nº 21/2025^(9)^ o MS organizou, em documento, orientações para a Rede Nacional de Atenção Integral à Saúde do Trabalhador e da Trabalhadora (Renastt) sobre como manejar o DVRT. Nele, o processo de Visat é apresentado em cinco etapas: 1) identificação do caso; 2) notificação compulsória no e-SUS Sinan;3) investigação epidemiológica para análise da relação com o trabalho e das situações de risco coletivas;4) monitoramento e análise contínua dos dados e das situações de risco; e 5) intervenção sobre os fatores de exposição, condicionantes e determinantes ambientais, organizacionais e psicossociais do agravo.

Com os dados alimentados numa plataforma única (e-Sus Sinan), monitorados e analisados continuamente, haverá maior clareza sobre as situações e locais onde o risco se apresenta, sendo propostas intervenções que venham promover a saúde vocal e prevenir as disfonias dos/as trabalhadores/as.

Vale destacar que qualquer profissional da saúde, dentre eles o/a fonoaudiólogo/a, pode e deve notificar o DVRT, inclusive os serviços privados. Caso a clínica não possua registro no Cadastro Nacional de Estabelecimentos de Saúde (CNES), poderá se associar, mediante autorização, a serviço cadastrado^(18)^. Para tanto, é necessário acessar o e-SUS Sinan^(19)^ e solicitar o cadastro, identificando o perfil adequado e incluindo uma justificativa^(20)^. No recente Webinário^(18)^, o MS orientou, passo a passo, como realizar o cadastro e a notificação do DVRT na plataforma^(18)^.

Além do registro histórico sobre o reconhecimento do DVRT, é importante que o/a leitor/a, principalmente o/a fonoaudiólogo/a, compreenda como esses avanços podem vir a mudar sua atuação, seja quanto aos procedimentos de avaliação de um/a trabalhador/a com queixa vocal, à distinção entre um DV comum e um DVRT, e à importância da notificação que, caso não seja realizada, provocará escassez de dados confiáveis para subsidiar políticas públicas. Para embasar esse conhecimento, sugerimos a leitura de referências que podem detalhar questões concernentes ao reconhecimento do DVRT^(6,21,22)^, ao seu entendimento no campo da Saúde do/a Trabalhador/a^(4,23)^ e à sua compreensão, segundo a Classificação Internacional da Funcionalidade (CIF)^(24)^.

Embora felizes com as conquistas, temos importantes desafios pela frente, como a implementação da rede de cuidado do DVRT, para a qual precisamos da participação e do engajamento dos/as fonoaudiólogos/as, sejam eles/elas da rede pública ou do serviço privado. Contamos com vocês nessa empreitada!
